# Spontaneous coronary artery dissection: clinical implications and diagnostic challenges. Overlooked and underappreciated in Asia?

**DOI:** 10.1002/clc.23484

**Published:** 2020-10-20

**Authors:** Ting‐Ting Low, Marie Houdmont, Hui W. Sim, Koo H. Chan, Poay H. Loh, Joshua P. Loh

**Affiliations:** ^1^ National University Heart Centre Singapore Singapore; ^2^ National University Hospital Singapore Singapore

## Abstract

Over the last decade, spontaneous coronary artery dissection (SCAD) has garnered much attention as a significant cause of acute coronary syndrome (ACS) and sudden cardiac death in women without classic cardiovascular risk factors. SCAD has been mostly studied in the West, with little recognition in Asia leading to under‐diagnosis and under‐representation. In this review, we highlight two distinct cases occurring at our center in Singapore, affecting two Singaporean women of Malay and Chinese descent. These 2 cases highlight that pregnancy‐associated SCAD is neither the most common nor only manifestation of SCAD. Through review of the literature, we emphasize the heterogeneity in case presentation paying particular attention to SCAD and its association with connective tissue disorders such as fibromuscular dysplasia. SCAD remains a diagnostic challenge for many cardiologists, here we shed light and dispel myths surrounding coronary angiography and review the use of intracoronary imaging. The successful treatment of this unique group of patients requires a high index of suspicion, and management within a multidisciplinary team. The development of a recovery program with access to support groups, allied health, and cardiac rehabilitation is paramount in improving outcomes for these patients in the long term. Further research and studies in our Asian population will help to enhance our understanding of this disease and develop practices to best manage our patients.

## 1 INTRODUCTION

Spontaneous coronary artery dissection (SCAD), once thought to be the veritable unicorn on coronary angiogram, has gained a significant amount of attention in the last decade. Previously labeled to be a rare condition, SCAD has now emerged as an important cause of acute coronary syndrome (ACS) and sudden cardiac death, especially in young and otherwise healthy women without apparent cardiovascular risk factors. SCAD is defined by atraumatic spontaneous dissection of an epicardial coronary artery, which is not related to atherosclerosis or iatrogenic injury. Myocardial injury most frequently results from luminal obstruction by a growing intramural hemorrhage that may be spontaneous or secondary to an intimal tear. Studies have reported the incidence to be between 1.1% and 4% of all ACS,^1^, ^2^ and as a cause of 15% and 35% of ACS in women under 50 years old.^3^, ^4^, ^5^ Remarkably, the advances in our understanding of SCAD was to a large extent driven by social media in the West, where stricken female patients found each other online, sharing sensational stories of their misdiagnosis and started advocating for the study of this under‐recognized disease. In this review, we illustrate two recent cases encountered at our center with distinct clinical entities and hope to raise awareness of this condition among the medical community, especially in the Asian region where SCAD research is sorely lacking compared to the West.

## 2 CASE 1—PREGNANCY‐ASSOCIATED SCAD

**FIGURE 1:**
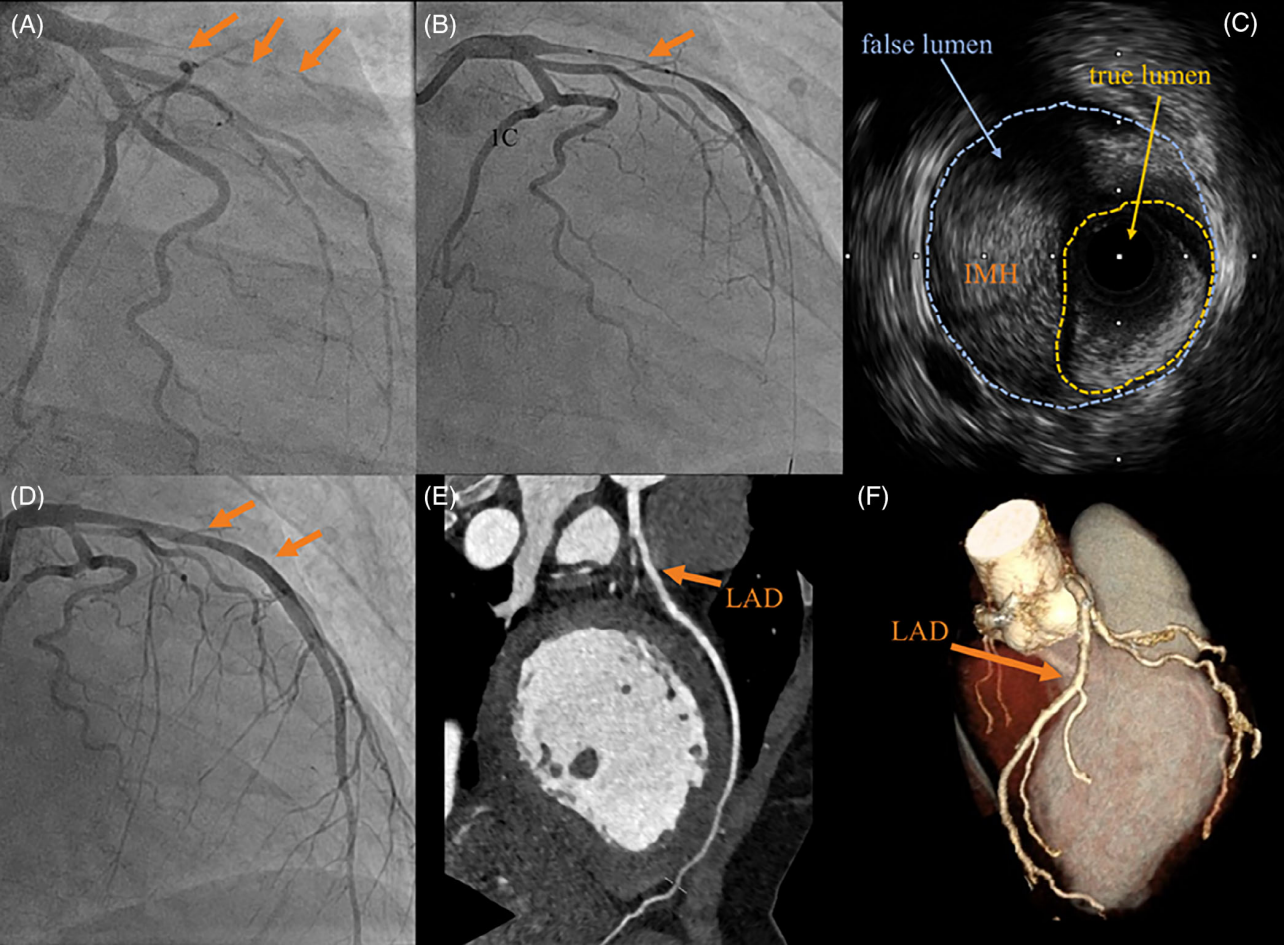
(A, B) Angiography showing left anterior descending artery (LAD) dissected with no flow on initial presentation, and then some flow return with gentle balloon angioplasty. (C) Intravascular ultrasound of the same LAD revealed the intramural hematoma (IMH) compressing the true lumen. (D) Angiogram showing good final results after angioplasty, no stent implanted. (E, F) Follow‐up computed tomography showed 0 calcium score and no significant stenosis of the LAD (arrows depict site of previous dissection)

At 3 weeks postpartum, a 32‐year‐old Malay Singaporean woman awoke with severe chest pain and diaphoresis. She arrived at the hospital emergency department 80 minutes after symptom onset, and the electrocardiogram showed ST‐segment elevation in the anterior leads. Her blood pressure at the Emergency Department (ED) triage was 155/96 mm Hg, heart rate of 74 beats per minute and oxygen saturations of 98% on room air. She was diagnosed with an acute ST‐segment elevation myocardial infarction (MI) and was loaded with aspirin and ticagrelor. She developed ventricular fibrillation en‐route to the cardiac catheterization laboratory, requiring defibrillation thrice. Emergent coronary angiography showed occlusion at the proximal left anterior descending (LAD) artery (Figure 1A). Thrombus aspiration was attempted without success. Balloon angioplasty was performed to the site of the occlusion with a 2.0 mm balloon which established antegrade TIMI II flow, and the underlying coronary lesion appeared to be consistent with a long SCAD (Figure 1B). This was confirmed on intravascular ultrasound (IVUS), which showed subintimal hematoma without clear breach in endothelium (Figure 1C). Further balloon angioplasty was performed with a larger 3.0 mm balloon and a 3.5 mm cutting balloon. Final angiography showed normal TIMI III flow without significant residual stenosis (Figure 1D). The rest of the coronary arteries appeared healthy with no evident features of atherosclerosis on angiography and IVUS.

Her left ventricular ejection fraction was later found to be severely impaired at 30%. Her cardiac troponins peaked at >22 500 ng/L. Her hemoglobin was within the normal range at 12.8 g/dL, and her creatinine was normal at 58 μmol/L. Multiparity was her main risk factor for SCAD; she had had six previous pregnancies, one miscarriage, and five live births. Her body mass index (BMI) was 21.6 kg/m^2^. Her cardiovascular risk factors included smoking and newly diagnosed hyperlipidemia (low‐density lipoprotein‐cholesterol 8.8 mmol/L, total cholesterol 11.0 mmol/L). A full body computed tomography angiogram did not reveal any extracoronary arteriopathy. She was discharged well on day 5 of admission. She had to stop breastfeeding due to her cardiac medications which include aspirin, rosuvastatin, ezetimibe, bisoprolol, and perindopril. She was also counseled against further pregnancies and was recommended to be on strict contraception.

She was followed‐up in the women's heart health clinic dedicated for patients with SCAD. Despite having to care for her six children, she found time to actively participate in our cardiac rehabilitation program. After 6 months of targeted heart failure therapy, her left ventricular ejection fraction improved to 45%. A follow‐up computed tomography coronary angiogram revealed a calcium score of 0, with mild luminal irregularity of her LAD artery (Figure 1D,E). She was taken off aspirin after completing 1 year of single antiplatelet therapy. The rest of her medications given on discharge remained.

### 2.1 Written consent

Written informed consent for patient information and images to be published was provided by the patients for this review.

## 3 CASE 2—NONPREGNANCY‐ASSOCIATED SCAD

**FIGURE 2:**
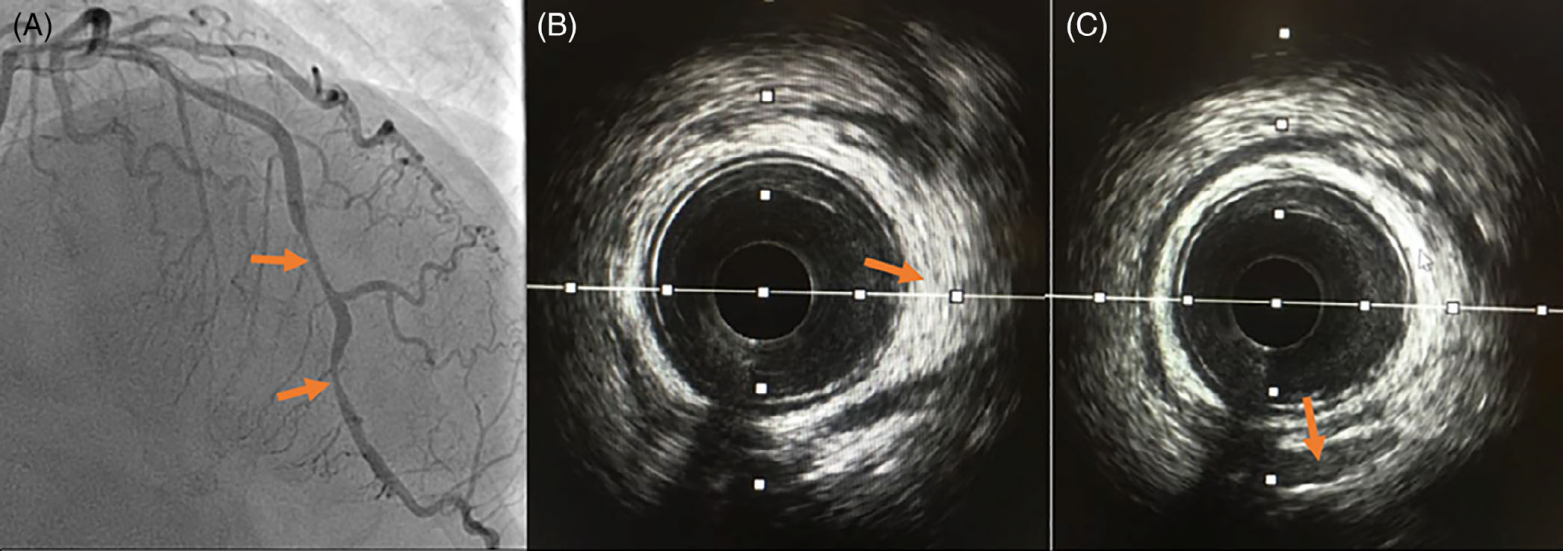
(A) Angiography showing narrowing of left anterior descending artery (arrowed). (B, C) Intravascular ultrasound of the same coronary artery showing intramural hematoma (echogenic signal) and dissection plane (echolucent signal) respectively, as depicted by arrows

A 46 years old Chinese Singaporean woman complained of two episodes of central chest pain at rest, 3 hours apart, with increasing intensity. At ED triage her blood pressure was 138/78 mm Hg, heart rate of 89 beats per minute and oxygen saturation of 98% on room air. Her initial electrocardiogram looked normal, but her troponin I level was raised to 1465 ng/L. Her laboratory investigations revealed a normal hemoglobin of 14.1 g/dL, and creatinine of 41 μmol/L. She was diagnosed with a non ST‐segment MI and was loaded with aspirin and ticagrelor. Coronary angiography revealed two tandem high‐grade stenotic lesions in the mid and distal LAD artery (Figure 2A). IVUS showed mild fibrotic plaques with significant intramural hematoma causing intraluminal narrowing (Figure 2B). Balloon angioplasty was performed with a 2.0 mm balloon, as there was on‐going angina, this was followed by implantation of a 2.25 × 33 mm drug‐eluting stent across the lesions to prevent re‐occlusion, with good result on IVUS. Her left ventricular function was preserved on echocardiogram. Her cardiovascular risk factors included newly diagnosed hyperlipidemia (low‐density lipoprotein 3.36 mmol/L) and hypertension (140/80 mm Hg). Her BMI was 22.1 kg/m^2^. She was discharged the following day with aspirin, ticagrelor, atorvastatin, bisoprolol, and lisinopril.

After 2 days, she was readmitted for recurrent chest pain that coincided with onset of her menstrual cycle. The initial concern was whether she had progression or recurrence of SCAD but her electrocardiogram was normal and the troponin I level was not higher than before. Her full body computed tomography angiogram did not reveal extracoronary arteriopathy. She was discharged the following day with spontaneous resolution of her chest pain. On follow‐up, she continued to experience recurrent chest pain episodes, and had to be reviewed frequently outpatient in the women's heart health clinic. Her electrocardiogram and troponin I level remained normal during each chest pain episode. She was treated with beta‐blocker and nitrate therapy. Eventually, her symptoms abated with completion of the cardiac rehabilitation program, which gave her confidence in performing her usual physical activities and exercise. She also received coaching from the occupational therapist on healthy lifestyle habits and stress management strategies. She completed 6 months of dual antiplatelet therapy, and was maintained on lifelong single antiplatelet agent. She remained on beta‐blockade, angiotensin converting enzyme‐inhibitor, and low dose statins.

## 4 PATHOPHYSIOLOGY AND TWO DISTINCT CLINICAL ENTITIES

**FIGURE 3:**
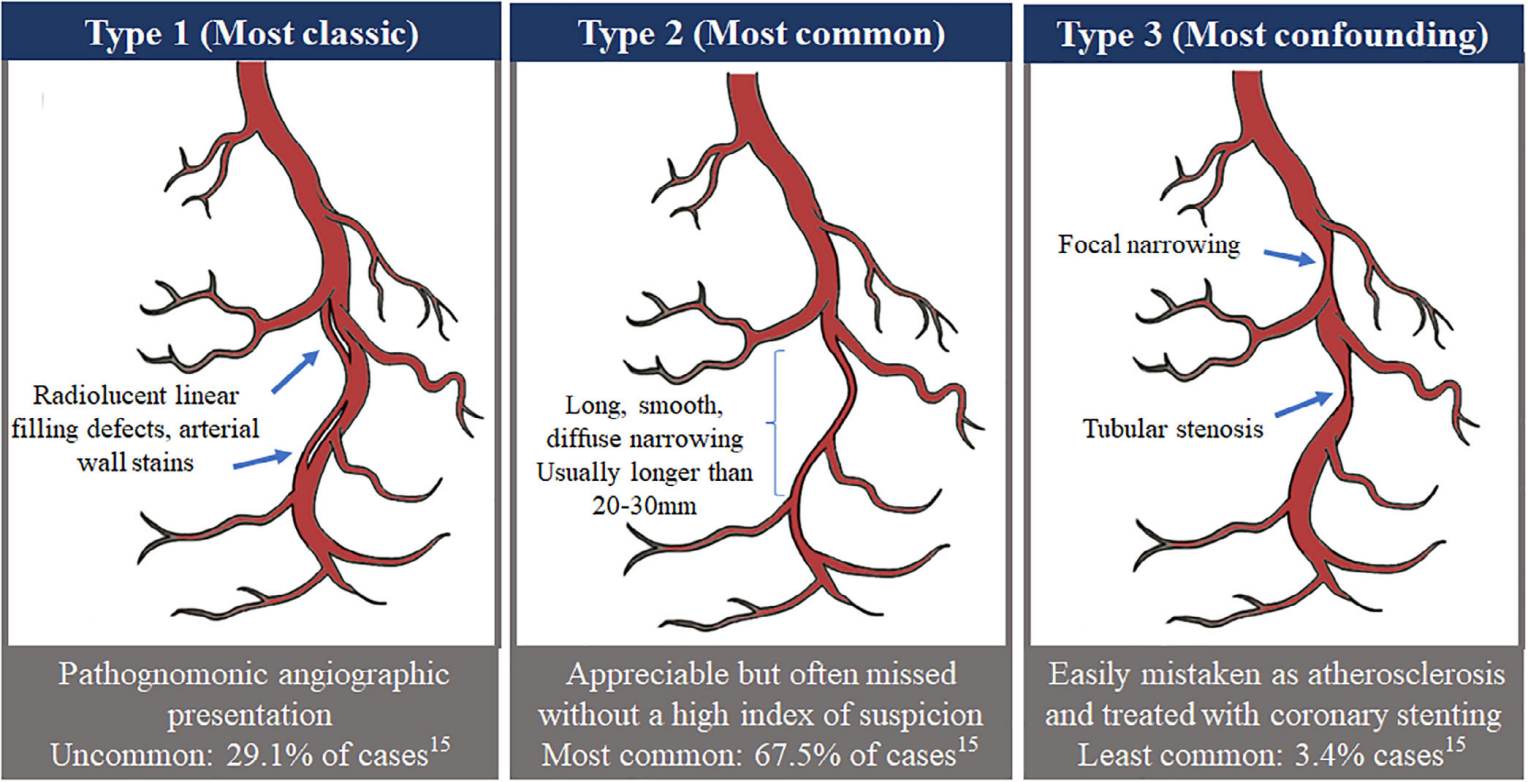
Angiographic SCAD classification

SCAD occurs as an impetuous tear of the coronary artery wall or separation of coronary artery wall layers, in the absence of atherosclerotic disease. The development of an intimal‐medial flap, bleeding into the false lumen or collection of intramural hematoma can cause intraluminal narrowing and obstruct coronary flow (see Figure 3), causing ischemia and ACS. Predisposing factors and associated conditions with SCAD are shown in Table 1.

**TABLE 1:** Consider a diagnosis of SCAD in the following clinical phenotype

Myocardial infarction in young women (<55 years of age) Absence of typical cardiovascular risk factors Especially if in peripartum state, and is multiparous Perimenopausal state or recent treatment with hormone replacement therapy History of fibromuscular dysplasia or connective tissue disease Recent extreme emotional or physical stressor Coronary angiogram without apparent culprit obstructive lesions

Historically, cardiologists picked up SCAD when it presents in its most dramatic form—usually a young pregnant woman with an acute ST‐segment elevation MI without known cardiovascular risk factors, such as in case 1. In the contemporary era with improved diagnostic approaches, it is now understood that SCAD most commonly presents as MI (with or without ST‐segment elevation) at a mean age of 51 ± 10 years,^2^ such as in case 2. In fact, recent cohort studies revealed that up to 55% are in the postmenopausal age group.^2^, ^5^ In addition, up to 10% of SCAD patients were found to be taking oral contraceptive or hormonal replacement therapies suggesting an important role of hormonal factors in SCAD pathophysiology.^6^ SCAD almost exclusively affects women, more than 90% of the time.^3^, ^7^, ^8^, ^9^, ^10^

Although chest pain remains the most prevalent clinical presentation, SCAD patients may develop a wide variety of symptoms. This includes life‐threatening ventricular arrhythmias such as ventricular fibrillation or ventricular tachycardia in up to 7.2% of patients. Back pain was reported in as many as 13.9%, and syncope in 0.5% of patients presenting with SCAD.^11^ SCAD has two distinct clinical entities, pregnancy‐associated SCAD (P‐SCAD) or nonpregnancy‐associated SCAD.

## 5 PREGNANCY‐ASSOCIATED SCAD

SCAD has been described to be the main etiology for MI in pregnancy, instead of garden‐variety atherosclerotic disease. The term P‐SCAD encompasses women presenting in pregnancy and the postpartum period up to 6 months. In fact, it occurs most frequently in the first week postdelivery.^12^ Although P‐SCAD remains rarer it is often more severe than nonpregnancy‐associated SCAD, resulting in multivessel dissections and acute heart failure, just as in our two contrasting cases above. Multiparity and infertility treatments are found to be additional risk factors for P‐SCAD.^12^ The exact mechanism of how pregnancy causes P‐SCAD is unclear. On histology, the pregnant state itself has been implicated as a contributor to arterial degeneration.^13^ It has also been proposed that female hormones responsible for the changes in connective tissue during pregnancy may contribute to intracoronary vessel fragility. The vascular endothelium contains estrogen and progesterone receptors,^14^ and is likely affected by dramatic changes of estrogen and progesterone levels that peak at term of pregnancy and dip postpartum. In addition, the hemodynamic changes associated with pregnancy may add shear stress on already vulnerable coronary vasculature. These include tachycardia, expansion of circulatory blood volume, increased cardiac output, increased cardiac requirements during labor, and massive fluid shifts with delivery. Interestingly, cases of SCAD have been reported to occur during lactation, further emphasizing the important role of hormones in the mechanism of P‐SCAD.^12^

## 6 NONPREGNANCY‐ASSOCIATED SCAD

With regards to SCAD that is not pregnancy‐related, the average age of women affected ranges from 45 to 53 years old. ^1^ Nonpregnancy‐associated SCAD now contributes a greater proportion of overall SCAD cases than P‐SCAD, the latter representing only up to 10% of SCAD cases in some studies.^12^ This is likely due to improved detection of cases in nonpregnant women. It is reported to be strongly associated with fibromuscular dysplasia (FMD). ^15^, ^16^, ^17^ FMD is a nonatherosclerotic, noninflammatory form of angiopathy—abnormal cellular growth in the vessel wall results in stenoses, aneurysms, and weakening of the vessel walls. It affects mostly medium‐to‐large arteries and can affect any arterial bed, including the coronary arteries. Among patients with identified FMD, renal involvement occurs in 60% to 75%, cerebrovascular involvement in 25% to 30%, visceral involvement in 9%, and arteries of the limbs in about 5%.^1^, ^2^ In SCAD patients with FMD, a typical “corkscrew appearance” due to arterial tortuosity were commonly found on angiography. SCAD associated with FMD is almost exclusively multifocal, suggesting an underlying vasculopathy. Multivessel SCAD is seen in up to 19% of patients.^15^ In case 2, we screened for but did not detect features to suggest FMD. In SCAD patients screened for FMD, concomitant extracoronary vascular abnormalities were frequently seen in the abdomen, pelvis, and neck arteries, and occurred in up to 50% of patients screened.^16^ Associations with vascular Ehlers‐Danlos syndrome, Marfan syndrome, adult polycystic kidney disease have been found but remain rare (<5% of cases).^18^ Family history, clinical presentation, and physical examination may help in selecting patients in which a genetic component is more likely, although routine genetic screening for all SCAD patients is not recommended.

Up until recently no clear genetic risk loci had been identified in SCAD, likely because of its rarity and its seemingly sporadic nature with only very few reported cases of inheritance. Just recently, a genetic link between SCAD and FMD has been described by Adlam et al. The genetic locus PHACTR1/EDN1 on chromosome 6 acting as a potential enhancer for endothelin‐1, has been associated with a variety of vascular diseases. Adlam et al have demonstrated that the variant risk allele rs9349379‐A at the PHACTR1 locus is more prevalent in both SCAD and FMD patients. Conversely the opposite allele rs9349379‐G confers greater risk of coronary artery disease.^19^

This work further highlights the importance of endothelins in the pathogenesis of SCAD. This is seen particularly when examining the interplay between estrogen and endothelin‐1 (ET‐1), which may partly explain SCAD's largely female preponderance. Estrogen withdrawal (eg, in the perimenopausal period) stimulates ET‐1 formation which is known to cause prolonged vasospasms potentially triggering coronary wall injury, hematoma formation, and dissection.^20^ At the molecular level, inductors of ET‐1 such as angiotensin II have been shown to cause elastin fragmentation, vascular smooth muscle cell disruption which results in an arterial wall prone to dissection,^20^ thereby demonstrating the key role ET‐1 plays in vascular injury. Further research targeted at ET‐1 levels may prove useful in the development of therapeutics for SCAD.

Extreme emotional or physical stress preceding SCAD had been reported in up to 40% of cases.^3^ Intense exercise such as lifting weights, vomiting, straining with bowel movement, and ingestion of methamphetamines and cocaine has also been associated with SCAD events. Emotional triggers were most often related to job stress, relationship difficulties, and death in the family.^15^ Addressing mood, and management of stressors during long‐term follow‐up is therefore essential. Nevertheless, in both our illustrated cases, the patients reported no identifiable extreme stressor that could have precipitated the ACS.

## 7 ANGIOGRAPHY AND INTRACORONARY IMAGING IS THE CRUX IN DIAGNOSIS

The diagnosis of SCAD is clinched on coronary angiography with three distinct angiographic patterns^21^ (Figure 3). While the pathognomonic finding of a dissected intimal plane on initial angiography is most readily recognized, this is not the most common angiographic appearance. Referred to as type 1 SCAD, this occurs in 26% to 43% of cases.^4^, ^15^, ^21^ Contrast staining of the arterial wall and multiple radiolucent lumens is usually considered diagnostic. Type 2 SCAD, where an intimal hemorrhage is present without intimal tear, is the most common finding in 55% to 78% of cases.^3^, ^4^, ^15^ Typically, angiography reveals a long obstructive or tapering vessel. Lastly, type 3 SCAD with discrete focal lesion(s) corresponding to intramural hematoma, mimics the appearance of atherosclerotic disease and easily leads to misdiagnosis.

When examining the coronary anatomy of SCAD patients there is often absence of coexistent coronary atherosclerosis where unaffected vessels are usually normal. Increased coronary tortuosity has been described, while multivessel SCAD has been reported in 13% of cases.^6^ In the same series, the predominant artery affected was the LAD artery (52%), followed by the LCx (37%) and RCA (23%).^6^

Intracoronary imaging is a useful adjunct to clarify the diagnosis especially in type 2 and 3. The meticulous use of IVUS and optical coherence tomography (OCT) has led to a burgeoning number of diagnoses of SCAD, in centers where this condition is well recognized. In fact, it is postulated that SCAD often goes under‐diagnosed in centers where there is lack of physician familiarity with this disease entity and a low index of suspicion for this condition. Both methods of intravascular imaging enable precise measurement of the extent of dissection. OCT has superior spatial resolution to IVUS in visualization of the intramural hematoma, intimal tears, and identification of the true lumen. Care must be taken when cannulating and intervening in the fragile coronaries for intracoronary imaging, so as not to further injure the vessels particularly when using Optical coherence tomography (OCT). The latter requires high intraluminal pressure contrast injections thereby potentially extending the dissection further. IVUS has the advantage of deeper penetration to assess the extent of intramural hemorrhage but its poorer resolution makes detecting intimal tears more difficult. Both IVUS and OCT are limited when SCAD is suspected in distal vessels. When in doubt, cardiac magnetic resonance imaging to confirm an endocardial infarct may be useful. Sometimes, diagnosis is made retrospectively on coronary angiogram after the cardiac magnetic resonance imaging confirms the presence of the infarcted territory.

There is a paucity of research on SCAD around Asia. Other than two sizable Japanese cohort studies, most published literature from Asia are case reports.^3^, ^5^ It is likely that SCAD goes under the radar without a willing mindset to perform intracoronary imaging in instances where there are disputable angiographic findings or in the setting of ACS without the usual cardiovascular risk factors. Type 2 SCAD may be mistakenly dismissed as “small vessel” disease or even “normal” coronary arteries to the untrained eye. Type 3 SCAD may be treated with stenting unknowingly since its angiographic appearance mimics atherosclerotic disease. The lack of resources to perform intracoronary imaging is a particularly challenging problem where there may be situations of inequitable healthcare access around the region. Large nonacademic cardiac centers, often driven by patient volume for service, may also not have the luxury of time during cardiac catheterization for extra diagnostic intravascular imaging. Suffice to say, SCAD has not garnered enough attention in Asia among cardiologists, and its prevalence is likely understated in this part of the world.

## 8 TREATMENT OF SCAD IS NOT SIMILAR TO USUAL CORONARY ARTERY DISEASE

A conservative approach in the management of SCAD is preferred when feasible, especially when there is TIMI II flow (partial perfusion) and above. Spontaneous healing of the dissected vessel is seen in the majority of cases when reassessed angiographically after weeks to months following the index episode. Percutaneous coronary intervention (PCI) should be considered if there is severe ongoing ischemia or extensive myocardial involvement. The vascular fragility in this subset of patients means that coronary stenting may be perilous with higher rates of intervention failure of up to 50%.^22^ Stent placement can cause “toothpasting” of the intramural hematoma, propagation of dissection, and occlusion of blood flow to major side branches. The incidence of iatrogenic injury during angiogram in SCAD cases was found to be 3.4% which may result from guidewire passage into the false lumen leading to secondary iatrogenic dissection. This is much higher than in non‐SCAD patients and may be associated with significant dissection with a mean dissection length of 45.6 mm.^23^ One study reported significantly increased rates of in‐hospital major adverse cardiovascular events (MACE) with 25% recurrent MI in the intervention group vs 5% in the conservative group.^23^ Coronary artery bypass graft surgery was ultimately required in a small number of patients where emergent revascularization is needed and PCI is deemed too complex. A recent large multicenter Canadian study of 750 SCAD patients revealed that 84.3% were treated conservatively, 14.1% underwent PCI, and 0.7% had coronary artery bypass graft surgery.^6^ However, in centers where SCAD is not readily appreciated, PCI rates are not predictable and may be higher.

There is currently no guideline directed medical therapy for SCAD. However, there is expert consensus that since the pathophysiology and disease progression differ greatly, medical treatment of SCAD cannot be extrapolated from treatment of usual atherosclerotic coronary disease. The routine use of heparin and dual antiplatelet therapy may result in increased intramural bleeding and further complications in SCAD patients. The recommendation is to give single agent antiplatelet therapy instead, and continuation beyond 1 year should be made on a case‐by‐case basis, taking into account the bleeding risk and benefits of therapy. Dual antiplatelet therapy is indicated if SCAD patients had coronary stents implanted to prevent stent‐related thrombosis. Regarding anticoagulation, heparin may be used during revascularization with PCI if indicated. However, long‐term anticoagulation therapy is recommended only where there is a clear clinical indication such as left ventricular thrombus formation or atrial fibrillation. Statin therapy should not be routine in SCAD and should be initiated according to guideline‐based indications for primary prevention of atherosclerosis. Beta‐blockers for those with depressed left ventricular function, hypertension, or arrhythmias have been shown to be helpful. Lastly, antianginals have been shown to have a role in symptomatic treatment of chest pain syndromes post‐SCAD, but routine use is not required in asymptomatic patients.^1^

Recurrent de novo SCAD has been reported in 10% of patients. A 2‐fold increase of recurrent SCAD is seen in those with hypertension. Secondary prevention with beta‐blocker should be considered. By reducing arterial shear stress, hypertension, and myocardial contractility beta‐blockers seem to play a pivotal role in protecting patients from further coronary dissection. Management of emotional and physical stressors through tailored exercise programs and psychosocial counseling also help. Illicit drug use of methamphetamine and cocaine should be addressed. Further pregnancy after a diagnosis of SCAD has usually been discouraged by the medical community, although this life changing advice has been based on few case reports and series.^10^

Cardiac rehabilitation is fundamental in recovery. Patients reported less chest pain and improved quality of life scores in depression, anxiety and stress level at the end of a dedicated rehabilitation program.^24^ There was also lower MACE rates in rehabilitation participants (4.3%) vs the nonparticipating group (26.2%). Long‐term prognosis is usually good for those who survive the initial episode, although the risk of recurrence has been reported to be up to 22%.^3^ Pregnancy is generally discouraged in patients diagnosed with SCAD, and strict contraceptive advice should be given if the affected women are in the reproductive age group.

## 9 CONCLUSION AND CALL FOR ACTION

A high index of suspicion is required to establish SCAD as a diagnosis on presentation (Table 2). The opportunity to make a confirmatory diagnosis may be lost, if additional relevant intracoronary imaging is not performed by the unsuspecting interventionist. A missed diagnosis most likely occurs in a relatively young woman who presents with ACS in the absence of traditional cardiovascular risk factors—sometimes conveniently brushed off as type 2 MI of unclear etiology. Although the largest SCAD cohort studies were conducted in the United States, Canada, Italy, and Switzerland,^1^ it would be presumptive to conclude that SCAD is a predominantly Caucasian disease. In fact, the incidence was reported as 0.31% in a Japanese SCAD study,^3^ similar to 0.2% estimated in Western cohorts. Until there is improved awareness of SCAD and an actionable plan to pick up this disease, it is likely to remain under‐diagnosed and under‐recognized in and around the Asian region. The two cases illustrated depict the heterogeneity in clinical presentation, angiographic appearance, and course of action taken by managing physicians. More research and prospective studies are needed, especially in the Asian region, to elucidate our understanding of this disease entity and its best management strategies. At present, our center is participating in a USA registry (DSRB ref: 2017/00763).

**TABLE 2:** Take‐home messages

Lack of physician familiarity is cited the most common reason of under‐diagnosis. It is *not* just a white women's disease. The most common clinical presentation of SCAD is *not* pregnancy‐related. An obvious dissection plane is *not* the most common angiographic appearance. Intracoronary imaging is the gold standard for diagnosis. Conservative management is generally preferred over coronary stenting. Screen for extracoronary vascular abnormalities. When in doubt, consider cardiac magnetic resonance imaging to confirm presence of infarcted territory, sometimes SCAD diagnosis is made retrospectively on coronary angiogram after.

## CONFLICT OF INTEREST

The authors declare no potential conflict of interests.

## Data Availability

The data that support the findings of this study are available on request from the corresponding author. The data are not publicly available due to privacy or ethical restrictions.

## References

[1] Hayes SN , Kim ESH , Saw J , et al. Spontaneous coronary artery dissection: current state of the science: a scientific statement from the American Heart Association. Circulation. 2018;137:e523‐e557. 29472380 10.1161/CIR.0000000000000564 PMC5957087

[2] Mahmoud AN , Taduru SS , Mentias A , et al. Trends of incidence, clinical presentation and in‐hospital mortality among women with acute myocardial infarction with or without spontaneous coronary artery dissection. JACC Cardiovasc Interv. 2018;11:80‐90. 29248409 10.1016/j.jcin.2017.08.016

[3] Nakashima T , Noguchi T , Haruta S , et al. Prognostic impact of spontaneous coronary artery dissection in young female patients with acute myocardial infarction: a report from the angina pectoris–myocardial infarction multicenter investigators in Japan. Int J Cardiol. 2016;207:341‐348. 26820364 10.1016/j.ijcard.2016.01.188

[4] Rashid HNZ , Wong DTL , Wijesekera H , et al. Incidence and characterisation of spontaneous coronary artery dissection as a cause of acute coronary syndrome ‐ a single‐centre Australian experience. Int J Cardiol. 2016;202:336‐338. 26426273 10.1016/j.ijcard.2015.09.072

[5] Nishiguchi T , Tanaka A , Ozaki Y , et al. Prevalence of spontaneous coronary artery dissection in patients with acute coronary syndrome. Eur Heart J Acute Cardiovasc Care. 2016;5:263‐270. 24585938 10.1177/2048872613504310

[6] Saw J , Starovoytov A , Humphries K , et al. Canadian spontaneous coronary artery dissection cohort study: in‐hospital and 30‐day outcomes. Eur Heart J. 2019;40:1188‐1197. 30698711 10.1093/eurheartj/ehz007 PMC6462308

[7] Abreu G , Galvão Braga C , Costa J , Azevedo P , Marques J . Spontaneous coronary artery dissection: a single‐center case series and literature review. Rev Port Cardiol. 2018;37:707‐713. 29935775 10.1016/j.repc.2017.07.019

[8] McGrath‐Cadell L , McKenzie P , Emmanuel S , Muller DWM , Graham RM , Holloway CJ . Outcomes of patients with spontaneous coronary artery dissection. Open Heart. 2016;3:e000491 10.1136/openhrt-2016-000491. 27621835 PMC5013459

[9] Motreff P , Malcles G , Combaret N , et al. How and when to suspect spontaneous coronary artery dissection: novel insights from a single‐centre series on prevalence and angiographic appearance. EuroIntervention. 2017;12:e2236‐e2243. 27973331 10.4244/EIJ-D-16-00187

[10] Saw J , Humphries K , Aymong E , et al. Spontaneous coronary artery dissection: clinical outcomes and risk of recurrence. J Am Coll Cardiol. 2017;70:1148‐1158. 28838364 10.1016/j.jacc.2017.06.053

[11] Luong C , Starovoytov A , Heydari M , Sedlak T , Aymong E , Saw J . Clinical presentation of patients with spontaneous coronary artery dissection. Catheter Cardiovasc Interv. 2017;89:1149‐1154. 28244197 10.1002/ccd.26977

[12] Tweet MS , Hayes SN , Codis E , et al. Spontaneous coronary artery dissection associated with pregnancy. J Am Coll Cardiol. 2017;70:426‐435. 28728686 10.1016/j.jacc.2017.05.055

[13] Rajagopalan S , Nwazota N , Chandrasekhar S . Outcomes in pregnant women with acute aortic dissections: a review of the literature from 2003 to 2013. Int J Obstet Anesth. 2014;23:348‐356. 25223644 10.1016/j.ijoa.2014.05.001

[14] Sader MA , Celermajer DS . Endothelial function, vascular reactivity and gender differences in the cardiovascular system. Cardiovasc Res. 2002;53:597‐604. 11861030 10.1016/s0008-6363(01)00473-4

[15] Saw J , Aymong E , Sedlak T , et al. Spontaneous coronary artery dissection: association with predisposing arteriopathies and precipitating stressors and cardiovascular outcomes. Circ Cardiovasc Interv. 2014;7:645‐655. 25294399 10.1161/CIRCINTERVENTIONS.114.001760

[16] Prasad M , Tweet MS , Hayes SN , et al. Prevalence of extracoronary vascular abnormalities and fibromuscular dysplasia in patients with spontaneous coronary artery dissection. Am J Cardiol. 2015;115:1672‐1677. 25929580 10.1016/j.amjcard.2015.03.011

[17] Rogowski S , Maeder MT , Weilenmann D , et al. Spontanous coronary artery dissection: angiographic follow‐up and long‐term clinical outcome in a predominantly medically treated population. Catheter Cardiovasc Interv. 2017;89:59‐68. 26708825 10.1002/ccd.26383

[18] Kaadan MI , Macdonald C , Ponzini F , et al. Prospective cardiovascular genetics evaluation in spontaneous coronary artery dissection. Circ Genom Precis Med. 2018;11:e001933. 29650765 10.1161/CIRCGENETICS.117.001933

[19] Adlam D , Olson TM , Combaret N , et al. Association of the PHACTR1/EDN1 genetic locus with spontaneous coronary artery dissection. J Am Coll Cardiol. 2019;73:58‐66. 30621952 10.1016/j.jacc.2018.09.085 PMC10403154

[20] Barton M , Yanagisawa M . Endothelin: 30 years from discovery to therapy. Hypertension. 2019;74:1232‐1265. 31679425 10.1161/HYPERTENSIONAHA.119.12105

[21] Saw J . Coronary angiogram classification of spontaneous coronary artery dissection. Catheter Cardiovasc Interv. 2014;84:1115‐1122. 24227590 10.1002/ccd.25293

[22] Tweet MS , Eleid MF , Best PJM , et al. Spontaneous coronary artery dissection: revascularization versus conservative therapy. Circ Cardiovasc Interv. 2014;7:777‐786. 25406203 10.1161/CIRCINTERVENTIONS.114.001659

[23] Prakash R , Starovoytov A , Heydari M , Mancini GBJ , Saw J . Catheter‐induced iatrogenic coronary artery dissection in patients with spontaneous coronary artery dissection. JACC Cardiovasc Interv. 2016;9:1851‐1853. 27609262 10.1016/j.jcin.2016.06.026

[24] Chou AY , Prakash R , Rajala J , et al. The first dedicated cardiac rehabilitation program for patients with spontaneous coronary artery dissection: description and initial results. Can J Cardiol. 2016;32:554‐560. 26923234 10.1016/j.cjca.2016.01.009

